# Looking Beyond Inherited Cardiomyopathy: Reconsidering the Role of Genetics in Heart Failure

**DOI:** 10.1016/j.jacadv.2026.103197

**Published:** 2026-10-21

**Authors:** Kemar Brown, Yuri Kim

**Affiliations:** a Division of Cardiovascular Medicine, Massachusetts General Hospital, Mass General Brigham Heart & Vascular Institute, Boston, Massachusetts, USA; b Department of Genetics, Harvard Medical School, Boston, Massachusetts, USA; c Division of Cardiovascular Medicine, Brigham and Women’s Hospital, Mass General Brigham Heart & Vascular Institute, Boston, Massachusetts, USA; d Division of Genetics, Brigham and Women’s Hospital, Mass General Brigham, Boston, Massachusetts, USA

**Keywords:** cardiomyopathy, heart failure, genetic testing, genetics

Two patients sustain anterior myocardial infarctions of similar size and receive identical guideline-directed medical therapy. One achieves near-complete myocardial recovery; the other progresses to end-stage heart failure (HF) requiring transplantation. We typically attribute this divergence in outcomes to adherence, comorbidity, or chance. We rarely ask whether part of the answer was written in the genome long before the first symptom emerged.

Contemporary HF care is organized around identifying the etiology of myocardial injury and initiating guideline-directed medical therapy. Current genetic-testing pathways generally prioritize patients whose phenotype, age at presentation, or family history raises suspicion for inherited cardiomyopathy.^1^^,^^2^ Although pragmatic, this approach implicitly treats inherited variation as clinically relevant primarily when it is considered the proximate cause of disease, overlooking its broader role in shaping the response to myocardial injury, risk of adverse remodeling, and likelihood of recovery. Cardiovascular genetics has accordingly centered on dilated (DCM), hypertrophic, arrhythmogenic, and restrictive cardiomyopathy, transforming care through improved diagnosis, cascade screening, and risk stratification. The central question is whether genetic testing should extend beyond defining the primary etiology to include identification of inherited variants that modify HF across diverse etiologies.

In this issue of *JACC: Advances*, Lima et al^3^ address these questions using whole-genome sequencing data from 1,013 unrelated adults with HF and left ventricular ejection fraction (LVEF) ≤50% enrolled in the GENIUS-HF cohort.^4^ Rare variants across 123 ClinGen-curated cardiovascular genes were classified per American College of Medical Genetics and Genomics criteria, and local ancestry inference was used to examine variant distribution in an admixed population. Overall, 66 individuals (6.5%) carried a qualifying likely pathogenic or pathogenic (LP/P) variant considered causal according to the investigators’ prespecified criteria, whereas 77.7% carried at least 1 variant of uncertain significance (VUS). Yield was highest in idiopathic DCM (9.9%), and *TTN* truncating variants (TTNtv) accounted for roughly half of qualifying findings. LP/P variants were also identified in etiologies rarely considered for testing: chagasic (4.9%), ischemic (4.7%), and hypertensive (3.8%) HF. The cohort is more heterogeneous than a traditional cardiovascular genetics referral population, although its single-center origin, median LVEF of 30%, and substantial representation of chagasic HF limit its generalizability to the broader HF population.

The most potentially actionable finding is one that etiology-first triage might have missed. Ten individuals carried pathogenic *TTR* variants, including 9 with p.Val142Ile (formerly V122I), a variant enriched in African-ancestry populations, in a cohort with median African ancestry proportion of approximately 24%. None carried a diagnosis of cardiac amyloidosis at enrollment. One was subsequently diagnosed with transthyretin cardiac amyloidosis (ATTR-CM) after new symptoms prompted reassessment. In an era of tafamidis, acoramidis, and vutrisiran,^5^ identification of a pathogenic *TTR* variant can prompt targeted phenotypic reassessment and, when ATTR-CM is confirmed, consideration of disease-modifying therapy. The genetic finding alone, however, neither establishes cardiac amyloidosis nor demonstrates that ATTR-CM caused the presenting HF syndrome. Whether carriers with an alternative assigned etiology have unrecognized ATTR-CM or would benefit from amyloid-directed therapy remains unresolved, but the prevalence estimate offered here is a necessary first step toward answering these questions.

These results challenge the traditional dichotomy between “genetic” and “acquired” HF. Rather than representing mutually exclusive processes, inherited susceptibility and acquired injury may coexist or interact along a continuum. Identification of an LP/P variant in a patient with obstructive coronary disease or positive Chagas serology does not establish primary causation. Instead, it raises at least 3 possibilities: the variant modified the myocardial response to injury; a genetic cardiomyopathy coexisted with an acquired disease; or the originally assigned HF etiology was incomplete or incorrect. The present study cannot distinguish among these explanations. A second-hit architecture of this kind has been proposed in myocarditis and in peripartum, cancer therapy-induced, and alcoholic cardiomyopathy.^6^

That hypothesis, however, is framed by these data rather than tested by them, and 3 gaps deserve emphasis. First, the study lacks a comparison group. TTNtv occur in roughly 1% of unselected populations,^7^ and without ancestry-matched HF-free controls, excess burden over background cannot be established. Designating a TTNtv as responsible for HF in a patient with prior myocardial infarction is therefore an attribution rather than a demonstration, and the 3.8% to 4.9% yields in non-DCM etiologies should be read with that caveat. Second, the analysis is cross-sectional. GENIUS-HF was designed to link genomic and electronic health record data to outcomes, yet carrier status is not related here to clinical outcomes including remodeling trajectory, LVEF recovery, arrhythmic events, or survival. Third, although the investigators used whole-genome sequencing, their analysis was restricted largely to rare predicted coding and splice-altering variants in 123 selected cardiovascular genes and did not assess copy-number variants, larger structural variants, or noncoding variation. The analytical scope therefore more closely resembles an expanded gene-panel analysis than a comprehensive interrogation of the genome. This cohort is well positioned to test whether these variants modify remodeling, recovery, and clinical outcomes.

The study’s second contribution concerns representation. Representation of non-European ancestries in genomic databases has increased only slowly, and variant classification depends on population allele frequencies, case reports, segregation data, and functional evidence, all unevenly distributed across ancestries. This limitation of the evidence base available to the American College of Medical Genetics and Genomics framework is compounded by documented disparities in access to genetic testing. Consequently, patients from historically under-represented backgrounds more often receive inconclusive results despite undergoing testing.^8^ Lima et al. quantify this at the haplotype level: segments harboring VUS showed modest but highly significant enrichment for African and Native American local ancestry, with observed-to-expected ratios of approximately 1.1 and 1.3, whereas segments harboring LP/P variants showed no such skew. The enrichment is modest and may reflect unequal representation in population databases and in the evidence used for variant interpretation, rather than a true ancestry-specific excess of disease-associated variation. Nevertheless, the analysis cannot establish the mechanism of this enrichment definitively. Notably, the pattern emerged in a cohort whose median ancestry was predominantly European; self-reported ancestry alone may not capture such haplotype-level patterns.

Are we ready to implement routine, unselected genetic testing across the spectrum of HF? Not yet. The present study supports broader consideration of genetic evaluation, but it does not establish the clinical utility of universal testing. A 6.5% qualifying LP/P yield and high prevalence of VUS could create substantial interpretive and counseling burdens, although the presence of any VUS across 123 genes should not necessarily be considered clinically consequential. Although current guidelines incorporate genotype into arrhythmic risk assessment for pathogenic variants in genes such as *LMNA*, *FLNC*, *PLN*, and *DSP*,^9^^,^^10^ prospective evidence that genotype-guided management improves outcomes remains limited. Scaling would require affordable sequencing, counseling capacity, clinical integration, cascade-screening infrastructure, and protection against genetic discrimination. Ultimately, risk models may need to also combine rare variants, polygenic risk, epigenetic state, and environmental exposure.

What Lima et al have done is shift the question. The future of HF genetics may depend less on whether cardiomyopathy is inherited or acquired than on how inherited genetic susceptibility and the primary mechanism of myocardial injury coexist or interact to shape remodeling, recovery, and clinical outcomes. Studies like this one, conducted in populations historically underrepresented in genomic medicine, begin to provide the prevalence estimates and the evidence base that such a shift will require. The next step is to determine prospectively whether these variants cause an unrecognized cardiomyopathy, coexist with an acquired disease, or modify the myocardial response to injury, and whether distinguishing among these possibilities improves patient outcomes.

## Funding support and author disclosures

Dr Brown is supported by the Harvard Medical School CTSA K12 Program 10.13039/100006691through NIH/NCATS K12TR004381 and the Massachusetts General Hospital Physician Scientist Development Award. Dr Kim is supported by the 10.13039/100000050NIH/NHLBI via K08HL164885, by the Heart Failure Research Foundation via Emerging Leader Grants, and by the British Heart Foundation’s Big Beat Challenge award to CureHeart (BBC/F/21/220106). All other authors have reported that they have no relationships relevant to the contents of this paper to disclose.
